# A Novel Hybrid Promoter ARE-hTERT for Cancer Gene Therapy

**Published:** 2017

**Authors:** S. V. Kalinichenko, M. V. Shepelev, P. N. Vikhreva, I. V. Korobko

**Affiliations:** Institute of Gene Biology, Russian Academy of Sciences, Vavilova Str. 34/5, Moscow, 119334, Russia; MRC Toxicology Unit, University of Leicester, Leicester, UK

**Keywords:** hTERT promoter, ARE elements, oxidative stress, hybrid promoter, cancer gene therapy, tumor-specific promoter

## Abstract

describe a novel hybrid tumor-specific promoter, ARE-hTERT, composed of the human *TERT *gene promoter (hTERT) and the antioxidant response element (ARE) from the human *GCLM *gene promoter. The hybrid promoter retains the tumor specificity of the basal *hTERT *promoter but is characterized by an enhanced transcriptional activity in cancer cells with abnormal activation of the Nrf2 transcription factor and upon induction of oxidative stress. In the *in vitro *enzyme-prodrug cancer gene therapy scheme, ARE-hTERT promoter-driven expression of CD : UPRT (yeast cytosine deaminase : uracil phosphoribosyltransferase) chimeric protein induced a more pronounced death of cancer cells either upon treatment with 5-fluorouracil (5FC) alone or when 5FC was combined with chemotherapeutic drugs as compared to the hTERT promoter. The developed hybrid promoter can be considered a better alternative to the hTERT promoter in cancer gene therapy schemes.

## INTRODUCTION

Gene therapy is a strategy that is witnessing dynamic development; several drugs have already been approved for clinical use, many are undergoing various phases of clinical trials, and a vast number of drugs are under development at laboratories. Various approaches to provide specific activity of gene therapy agents in cancer cells have been proposed and validated, including post-transcriptional regulation of the therapeutic transgene expression level in cancer cells via the selective stabilization of the transcript in tumor cells [1] or destabilization of the transcript in normal cells [2]. Along with these relatively new approaches, activation of transgene expression predominantly in tumor cells by tumor-specific promoters remains one of the most frequently used, well-explored, and justified strategies to provide tumor specificity in cancer gene therapy [3].

Tumor-specific promoters have been successfully validated in many cases, but their usage is associated with several drawbacks, including a far from absolute tumor specificity and low promoter activity, which affects the transgene expression level and, consequently, the therapeutic effect. In particular, the human telomerase reverse transcriptase (hTERT) gene promoter is one of the best characterized tumor-specific promoters and is active in a wide variety of tumors, providing the advantage of targeting cancer cells of different origins [3-7]. Nevertheless, the hTERT promoter is relatively weak, which might affect the overall efficiency of the therapy in clinical settings. So, several attempts to improve its activity have been made. Since the hTERT promoter is “TATA-less,” two modifications were proved to increase promoter activity: linking the promoter to a synthetic TATA-box or to a minimal early/immediate cytomegalovirus promoter to provide conventional basal promoter elements [8, 9]. However, the activity of the hTERT promoter varies greatly among different tumor cell lines, which might compromise the advantage of its universality [10]. Therefore, taking into account the above considerations, there is a definite need to increase hTERT promoter activity in tumor cells while retaining its tumor cell specificity.

Many promoters like hTERT show tumor-specific activity due to reactivation in tumor cells, and the tumor specificity of transgene expression can be further increased by exploiting genetic regulatory elements that respond to a perturbed tumor microenvironment or are abnormally active due to somatic mutations in tumor cells. This strategy can be exemplified by exploiting *cis*-acting regulatory elements that provide a transcriptional response to oxidative stress or hypoxia, which are the hallmarks of many tumors. In particular, antioxidant response elements (ARE), the binding sites for the Nrf2 transcription factor, which is a master activator of the oxidative stress response, suffice to support tumor-specific transgene expression when linked to a basal promoter [11]. In such a setting, transcription is maintained due to the abnormal Nrf2 activation occurring in response to intrinsic oxidative stress in tumor cells or because of somatic mutations resulting in constitutive Nrf2 activation.

In this paper, we show that combining the tumor-specific hTERT promoter with ARE results in increased activity of the hybrid promoter in tumor cells compared to the hTERT promoter. At the same time, this modification did not affect promoter activity in non-cancerous cells, in which Nrf2 is not activated under normal conditions. This approach can be used to increase the transgene expression level and activity of therapeutic proteins in tumor cells without an appreciable loss of tumor specificity.

## MATERIALS AND METHODS

**Cell culture**

Human lung epidermoid carcinoma Calu-1 (ECACC #93120818), nonsmall cell lung carcinomas NCI-H1299 (ATCC #CRL-5803) and A549 (ATCC #CRL-185), and nonsmall cell lung bronchioalveolar carcinoma NCI-H358 (ATCC #CRL-5807) cell lines were cultured in a DMEM/F12 (1 : 1) medium (HyClone, USA) supplemented with 10% fetal bovine serum (HyClone), penicillin (100 U/ml), and streptomycin (100 µg/ml) (Gibco, UK). Human bronchial epithelial cells HBEpC (ECACC #502-05) were cultured in the Bronchial Epithelial Growth Medium (Lonza, Switzerland). For cell viability or luciferase reporter gene assays, cells were seeded into 24-well plates at an indicated density (NCI-H1299, 20000 cells/well; A549, 30000 cells/well; Calu-1, 40000 cells/ well; NCI-H358, 150000 cells/well; HBEpC, 80000 cells/ well) and transfected with a Unifectin-56 transfection reagent (Rusbiolink, Russia) the next day.

**Plasmids**

The plasmid phTERT-Luc encoding firefly luciferase under the control of a -206…+37 nt *hTERT *promoter was described earlier [10]. Plasmid pARE-hTERT-Luc containing firefly luciferase cDNA under the control of the hybrid ARE-hTERT promoter was generated by cloning 56 bp ARE (tgagtaacggttacgaagcactttctcggctacgatttctgcttagtcattgtctt) from the human glutamate-cysteine ligase modifier (*GCLM*) gene promoter to the 5’-end of the hTERT promoter in phTERT-Luc plasmid [12]. phTERT-CD : UPRT and pARE-hTERT-CD:UPRT plasmids for the expression of the yeast cytosine deaminase:uracil phosphoribosyltransferase (CD : UPRT) chimeric protein under the control of hTERT or ARE-hTERT promoter, respectively, were constructed on the backbone of pBluescriptII SK(- ) vector (Stratagene, USA) [13]. The SV40 signal for transcription termination and polyadenylation derived from pBK-CMV expression vector (Stratagene) was cloned at the 3’-end of CD : UPRT cDNA.

**Chemicals**

*Tert*-butylhydroquinone (tBHQ), doxorubicin, cisplatin, etoposide, and 5-fluorocytosine (5FC) were purchased from Sigma-Aldrich (USA).

**Luciferase reporter gene assay**

Cells were transfected in triplicate for each plasmid combination by a mixture of the firefly reporter plasmid (phTERT-Luc, pARE-hTERT-Luc or promoterless pGL3-Basic plasmid (Promega)) with pRL-CMV (Promega, USA) plasmid (encoding the *Renilla* luciferase reporter gene under the control of the CMV immediate early enhancer/promoter). If indicated, cells were treated with 100 µM tBHQ for 24 hrs prior to harvesting for luciferase activity analysis. Luciferase activities were quantified 2 days after transfection using the Dual-Luciferase® Reporter Assay System (Promega). Firefly luciferase activity was normalized to the *Renilla* luciferase activity, and the average values of relative light units (RLU) and standard deviation (SD) were calculated.

**Cell viability assay**

Cells were transfected with CD : UPRT-encoding plasmids or mock-transfected with the pBK-CMV vector and plated into the wells of a 96-well plate 24 hrs after transfection (2,000 cells/well for NCI-H1299, A549 and Calu-1 cell lines, and 5,000 cells/well for NCI-H358 cells). 5FC and/or etoposide, cisplatin, or doxorubicin were added 24 hrs after plating. Cell culture medium containing 5FC and/or chemotherapeutic drugs was changed for a fresh one after 24 and 96 hrs of incubation. If indicated, tBHQ was present in the medium at a concentration of 100 µM between 24 and 96 hrs of incubation. Cell viability was determined after 120 hrs of incubation using the CellTiter96® AQueous One Solution Cell Proliferation Assay (Promega) according to the manufacturer’s instruction. Each experimental point was analyzed in triplicate. Cell viabilities were normalized to the viability of cells incubated in the absence of 5FC and chemotherapeutic drugs, which was taken as 100%.

## RESULTS

**Design of the hybrid ARE-hTERT promoter**

Hurttila et al. previously analyzed the potency of AREs derived from several Nrf2-responsive genes to support transgene expression under oxidative stress conditions and showed that the highest expression level among the tested AREs was provided by ARE from the *GCLM *gene promoter [12]. Based on these findings, we used ARE from the human *GCLM *gene promoter and placed it at the 5’-end relative to the transcription start site of the -206…+37 nt fragment of the hTERT promoter, which is sufficient to support tumor-specific transcription [5].

**Activity of the hybrid ARE-hTERT promoter in cancer and normal cells**

**Fig. 1:**
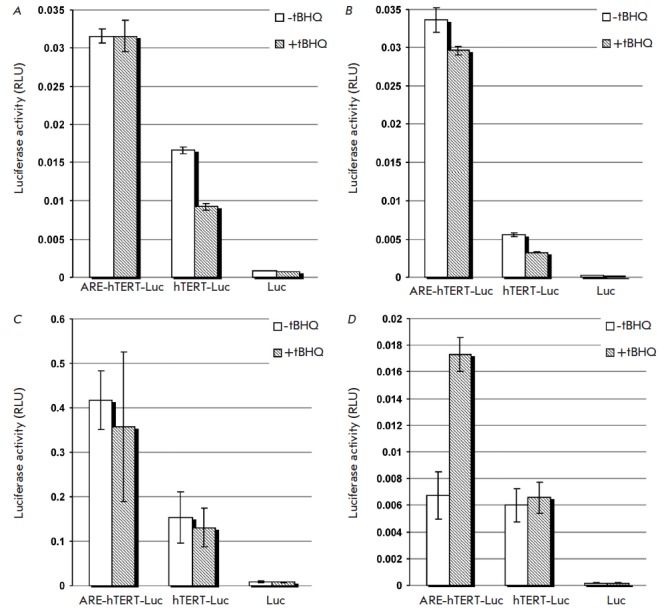
Effect of ARE-modification of the hTERT promoter on luciferase reporter gene activity in lung cancer cell lines. The luciferase reporter gene activity was measured in NCI-H1299 (**A**), Calu-1 (**B**), A549 (**C**), and NCI-H358 (**D**) cells transfected with phTERT-Luc (*hTERT-Luc*), pARE-hTERT-Luc (*ARE-hTERT-Luc*), or promoterless pGL3-Basic (*Luc*) plasmid, together with pRL-CMV plasmid for normalization. If indicated (*hatched bars*), cells were treated with 100 μM tBHQ for 24 hrs. The data are shown as average RLU values ± SD.

We used a luciferase reporter assay to compare the activities of the conventional and the hybrid hTERT promoters in cancer and normal cells. In three out of four tested lung cancer cell lines (NCI-H1299, Calu-1 and A549), the ARE-hTERT promoter showed 2- to 3-fold higher activity compared to the unmodified hTERT promoter, while in NCI-H358 cell line the introduction of ARE did not significantly improve the promoter activity (*Fig. 1* “-tBHQ” samples). Importantly, a similar lack of effect on the transcriptional activity of the hybrid promoter was observed in noncancerous HBEpC cells: relative luciferase activities in pARE-hTERT-Luc- and phTERT-Luc-transfected cells were 1.41 ± 0.45 and 1.00 ± 0.214 (*P *= 0.2272, two-tailed Student’s *t*-test). Therefore, the ARE-hTERT promoter outperformed the unmodified hTERT promoter in three out of four tested lung cancer cell lines, while modification did not affect the promoter activity in normal cells.

**Induction of oxidative stress can stimulate hybrid promoter activity**

Next, we studied if induction of oxidative stress could increase ARE-hTERT promoter activity in cancer cells. Treatment of NCI-H358 cells with tBHQ resulted in ~2.5-fold induction of luciferase reporter gene activity under the control of the ARE-hTERT promoter, while having no effect on the activity of the hTERT promoter (*Fig. 1*, “+ tBHQ” samples), which is well in line with the responsiveness of *GCLM *ARE to external oxidative stress [12]. At the same time, tBHQ treatment did not affect *ARE*-hTERT promoter activity in Calu-1, A549, and NCI-H1299 cell lines in which ARE-hTERT promoter activity significantly outperformed that of the unmodified hTERT promoter without an external oxidative stressor (*Fig.1*, “-tBHQ” samples). Taken together, the hybrid ARE-hTERT promoter possesses higher activity in cancer cells under basal conditions compared to the hTERT promoter, likely owing to the activation of the Nrf2 pathway that frequently occurs in cancer cells due to somatic mutations or the increased level of reactive oxygen species (ROS) [14-19]. In cells with low basal activity of the hybrid promoter, its transcriptional activity can be boosted by oxidative stress inducers.

**A hybrid promoter improves the efficiency of enzyme-prodrug suicide cancer gene therapy *in vitro***

**Fig. 2:**
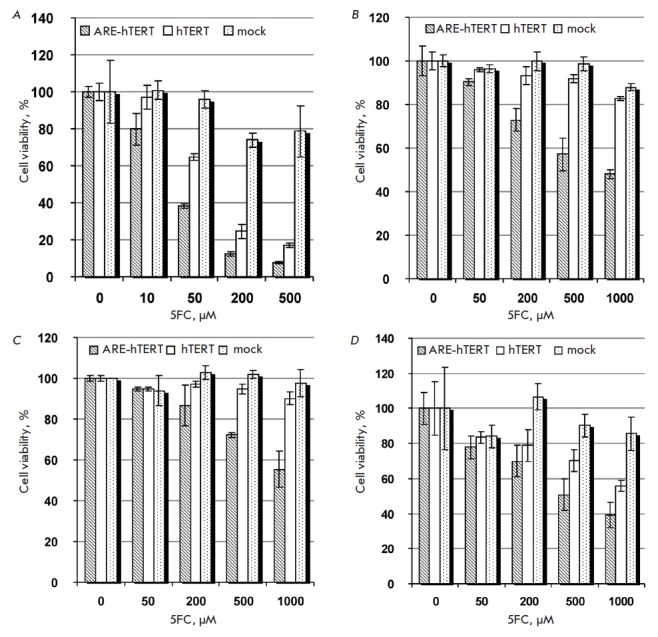
Comparison of the cytotoxic effects of *hTERT *and *ARE-hTERT *promoter-driven CD:UPRT expression in lung cancer cell lines in the presence of 5FC. Relative viabilities of NCI-H1299 (**A**), Calu-1 (**B**), A549 (**C**), and NCI-H358 (**D**) cells transfected with pARE-hTERT-CD : UPRT (*hatched bars*), phTERT-CD : UPRT (*blank bars*), and pBK-CMV (mock, *dotted bars*) plasmids after incubation with indicated concentrations of 5FC are shown as average values ± SD of the percentage of viable cells relative to the viability of similarly transfected cells incubated in the absence of 5FC.

**Fig. 3:**
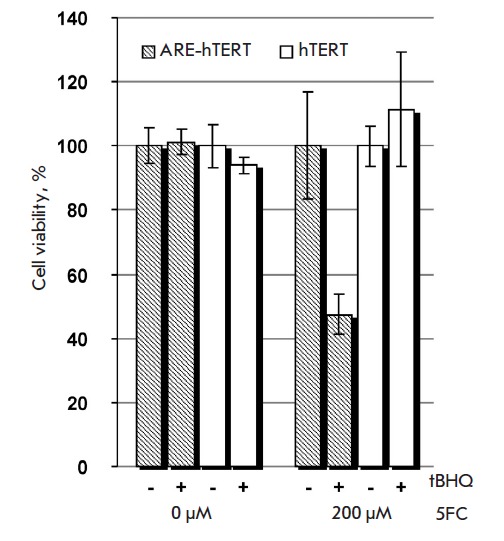
The cytotoxic effect of *ARE-hTERT *promoter-driven CD : UPRT expression in the presence of 5FC is enhanced by an oxidative stress inducer in NCI-H358 cells. NCI-H358 cells were transfected with pARE-hTERT-CD : UPRT (*hatched bars*) or phTERT-CD : UPRT (*blank bars*) plasmid, and cell viabilities were determined after incubation in the absence or presence of 200 μM of 5FC and/or 100 μM tBHQ as indicated. Data are shown as average values ± SD of the percentage of viable cells relative to the viability of similarly treated cells incubated in the absence of 5FC.

The increased activity of the hybrid promoter compared to the conventional hTERT promoter observed in NCI-H1299, Calu-1, and A549 cells in luciferase reporter gene assay leads one to assume that the modification of the hTERT promoter with ARE will also improve the performance of cancer gene therapy vectors. In order to directly address this issue, we compared the capacities to induce cancer cell death in the enzyme-prodrug CD : UPRT–5FC suicide cancer gene therapy scheme when CD : UPRT expression was driven by either an unmodified or ARE-modified hTERT promoter [20]. As expected, ARE-hTERT promoter-driven CD : UPRT expression resulted in a more pronounced level of cell death in the presence of the same 5FC concentrations compared to hTERT-driven expression (*Fig. 2*). Alike, in agreement with the results of a promoter activity analysis with the reporter gene, ARE modification of the hTERT promoter directing CD : UPRT expression did not affect cytotoxicity for NCI-H358 cells. At the same time, simultaneous treatment of NCI-H358 cells with tBHQ significantly augmented the cytotoxic effect only when the ARE-modified promoter was used (*Fig. 3*), while tBHQ did not increase cytotoxicity when the ARE-hTERT promoter was used instead of the unmodified promoter in NCI-H1299, Calu-1, and A549 cells, where the hybrid promoter is intrinsically more active than the hTERT promoter according to the reporter gene assay (data not shown).

**ARE-hTERT promoter-driven enzyme-prodrug suicide cancer gene therapy more efficiently sensitizes cancer cells to conventional chemotherapeutic drugs**

**Fig. 4:**
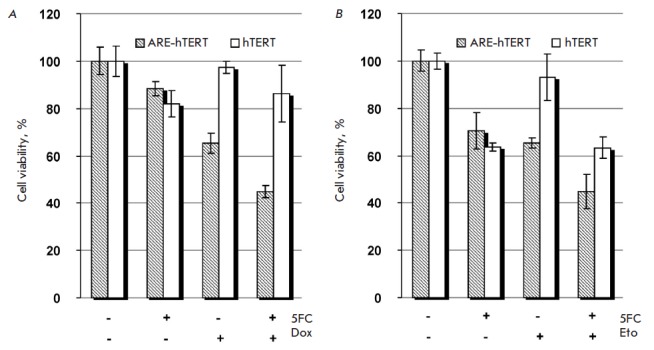
Comparison of the cytotoxic effects of *hTERT *and *ARE-hTERT *promoter-driven CD : UPRT expression in lung cancer cell lines in the presence of 5FC when combined with chemotherapeutic agents. NCI-H1299 (**A**) or A549 (**B**) cells were transfected with pARE-hTERT-CD : UPRT (*hatched bars*) or phTERT-CD : UPRT (*blank bars*) plasmid and incubated in the presence or absence (as indicated) of 10 μM of 5FC and 0.1 μM of doxorubicin (Dox) (**A**) or 500 μM of 5FC and 2 μM of etoposide (Eto) (**B**). Data are shown as average values ± SD of the percentage of viable cells relative to the viability of similarly treated cells incubated in the absence of 5FC and chemotherapeutic agents.

ARE-driven enzyme-prodrug suicide cancer gene therapy was reported to increase the sensitivity of cancer cells to chemotherapeutic drugs: in particular, to doxorubicin [11]. Therefore, we questioned if ARE*-*hTERT promoter-driven CD : UPRT-5FC enzyme-prodrug suicide cancer gene therapy would also result in enhanced cytotoxicity when combined with chemotherapeutic drugs. *Figure 4A* demonstrates that hTERT-driven CD : UPRT expression did not result in NCI-H1299 cell death in the presence of 10 µM 5FC. Also, treatment with 0.1 µM doxorubicin resulted only in marginal NCI-H1299 cell death. Notably, under the same settings, application of the ARE-modified hTERT promoter resulted in substantial cell death (~40%), which was further significantly potentiated by combined treatment with doxorubicin (*Fig. 4A*). Similar observations were made for A549 and Calu-1 cells treated with doxorubicin, etoposide or cisplatin (*Fig. 4B* and data not shown). Importantly, under our experimental settings, hTERT promoter-driven cancer gene therapy showed no effect alone and failed to potentiate the cell death elicited by chemotherapeutic drugs, while modification of the promoter with ARE resulted in pronounced cytotoxicity both in the case of cancer gene monotherapy and when it was combined with chemotherapeutic drugs.

## DISCUSSION

We tested the hypothesis that introduction of ARE into a promoter with intrinsic tumor specificity, which is routinely used to target cancer cells in cancer gene therapy, will enhance promoter activity without an appreciable loss of specificity toward tumor cells. Indeed, ARE linked to a nonselective minimal promoter was previously shown to provide tumor-specific expression owing to an aberrantly activated Nrf2 transcription factor [14-19] or intrinsically higher ROS levels in tumor cells [11, 12]. As we demonstrated, a hybrid promoter containing the human *TERT *gene promoter and ARE derived from the human *GCLM *gene promoter showed better performance in 3 of 4 tested cancer cell lines both in reporter gene assay and in the CD : UPRT-5FC suicidal cancer gene therapy scheme. In NCI-H358 cells, where hTERT promoter modification did not affect the promoter activity (which suggests a lack of abnormal Nrf2 regulation), the activity of the hybrid promoter could be boosted by oxidative stress inducers such as tBHQ. It is important to mention that an analysis in primary epithelial HBEpC cells showed a lack of any appreciable increase in promoter activity after the inclusion of ARE in the promoter, thus demonstrating that the introduced modification did not affect the cancer-cell specificity of transcription.

Our results indicate that the novel hybrid promoter, while retaining a high cancer cell specificity, will outperform the conventional hTERT promoter in a substantial proportion of tumors where Nrf2 is activated due to a somatic mutation. In addition, cancer cells are generally characterized by an increased ROS level both *in vitro* and *in vivo*, which is caused by several factors, such as altered metabolism and inadequate vascularization [21]. In addition, many conventional chemotherapeutic drugs are known to induce oxidative stress; therefore, combination of ARE-hTERT driven cancer gene therapy with conventional chemotherapeutic drugs *in vivo *might further potentiate the overall efficiency of the treatment through the promotion of therapeutic transgene expression.

The efficiency of a cancer gene therapy is primarily determined by the therapeutic transgene expression level, which should be high enough to elicit a therapeutic effect. In this work, we used the CD : UPRT-5FC enzyme-prodrug cancer gene therapy approach, in which the overall efficiency of the therapy is determined by the efficiency of plasmid delivery into cancer cells, promoter activity, and cell sensitivity to the cytotoxic agent obtained from the prodrug conversion. These parameters will obviously vary for specific cell types, potentially resulting in a loss of treatment efficiency. Indeed, under the experimental settings used (*Fig. 4*), the hTERT-driven gene therapy failed to result in tumor cell elimination or to enhance the cytotoxic effect of chemotherapeutic drugs. However, under identical conditions, modification of the hTERT promoter with ARE restored the cytotoxic effect of the gene therapy and significantly potentiated the chemotherapeutic drug-induced cytotoxicity. These results explicitly demonstrate that the application of a hybrid promoter, instead of the conventional hTERT promoter, would broaden the therapeutic efficiency of gene therapy, thus demonstrating the advantages of the reported hybrid promoter.

## CONCLUSIONS

We have created a novel tumor-specific promoter that retains the tumor specificity of the basal hTERT promoter but is characterized by an enhanced transcriptional activity in cancer cells due to either abnormal Nrf2 transcription factor activation or stimulation with ROS inducers. Owing to the above characteristics, the ARE-hTERT hybrid promoter can be considered a better alternative to the hTERT promoter in cancer gene therapy schemes. In addition, the combination of ARE with other tumor- or tissue-specific promoters used to develop vectors for cancer gene therapy can be regarded as a way to improve their performance without an appreciable loss of specificity.

## Abbreviations

ARE: antioxidant response element
CMV: cytomegalovirus
GCLM: glutamate–cysteine ligase modifier subunit
TERT: telomerase reverse transcriptase
tBHQ: tert-butylhydroquinone
ROS: reactive oxygen species
RLU: relative light units
SD: standard deviation
CD:UPRT: yeast cytosine deaminase : uracil phosphoribosyltransferase chimeric protein
5FC: 5-fluorouracil
